# The chromosomal genome sequence of the feather duster worm, *Sabellastarte* sp. h YS-2021 (Sabellida: Sabellidae) and its associated microbial metagenome sequences

**DOI:** 10.12688/wellcomeopenres.26503.1

**Published:** 2026-05-11

**Authors:** Yanan Sun, Keith Kei, Jian-Wen Qiu, José M. Martín-Durán, Graeme Oatley, Elizabeth Sinclair, Eerik Aunin, Noah Gettle, Camilla Santos, Michael Paulini, Haoyu Niu, Victoria McKenna, Rebecca O’Brien, Wellcome Sanger Institute Tree of Life Management, Samples and Laboratory team, Wellcome Sanger Institute Scientific Operations: Sequencing Operations, Wellcome Sanger Institute Tree of Life Core Informatics team, EBI Aquatic Symbiosis Genomics Data Portal Team, Aquatic Symbiosis Genomics Project Leadership

**Affiliations:** 1 Laboratory of Marine Organism Taxonomy and Phylogeny, Institute of Oceanology, Chinese Academy of Sciences, Qingdao, China; 2 Hong Kong Baptist University School of Continuing Education, Hong Kong, Hong Kong; 3 Hong Kong Baptist University Department of Biology, Hong Kong, Hong Kong; 4 Queen Mary University of London, London, England, UK; 5 Wellcome Sanger Institute, Hinxton, England, UK

**Keywords:** Sabellastarte sp. h YS-2021, feather duster worm, genome sequence, chromosomal, Sabellida, microbial metagenome assembly

## Abstract

We present a genome assembly from an individual *Sabellastarte* sp. h YS-2021 (feather duster worm; Annelida; Polychaeta; Sabellida; Sabellidae). The genome sequence has a total length of 1 786.39 megabases. Most of the assembly (97.94%) is scaffolded into 14 chromosomal pseudomolecules. The mitochondrial genome has also been assembled, with a length of 15.35 kilobases. From the metagenome data, we recovered 5 bins, of which one was a high-quality MAG.

## Species taxonomy

Eukaryota; Opisthokonta; Metazoa; Eumetazoa; Bilateria; Protostomia; Spiralia; Lophotrochozoa; Annelida; Polychaeta; Sedentaria; Canalipalpata; Sabellida; Sabellidae; *Sabellastarte*; unclassified *Sabellastarte*; *Sabellastarte* sp. h YS-2021 (NCBI:txid2886754).

## Background

*Sabellastarte* is a genus of Sabellidae living in self-secreted parchment tubes in tropical to temperate waters of the Caribbean Sea and Indo-Pacific ( Fauchald, 1977). They are usually large, with the tube measuring up to 1.5 cm in diameter and 20 cm in length. When extended, the radioles form a double fan-shaped crown. *Sabellastarte* species are commonly called feather duster worms. Because of their large size and the attractiveness of the radioles, *Sabellastarte* is commonly harvested for the aquarium trade ( Capa *et al.*, 2010). Some species of *Sabellastarte* live among crevices of reef-building corals, while others insert the lower part of their tubes into coarse substrates.

The genus *Sabellastarte* is defined by a combination of characteristics such as the absence of radiolar flanges, the presence of dorsal flanges on the base of the radiole crown, and a large collar with flanking pockets on both sides ( Knight-Jones & Mackie, 2003). However, some characteristics used to distinguish species show developmental variation ( Knight-Jones & Mackie, 2003). Based on morphological analysis, Knight-Jones & Mackie (2003) considered eight valid species of *Sabellastarte.* In an integrated morphological and molecular analysis, Capa *et al*. (2010) recognised only six lineages, each exhibiting a specific geographic distribution.

*Sabellastarte japonica* (Marenzeller, 1885) is widely distributed from Japan to northern Australia and Caledonia ( Knight-Jones & Mackie, 2003). Based on a comparison with the holotype from Nagasaki, Japan, Knight-Jones & Mackie (2003) considered the specimens collected from Hong Kong to be conspecific with *S. japonica.* However, they noted some differences, such as larger than the holotype and the radioles having narrower and closer pigment bands.

While searching for a representative species of the family Sabellidae for genome sequencing, we collected *Sabellastarte* specimens from Yau Tong, Hong Kong at a depth of 6 m on a coarse bottom within a fringing coral community ( Yeung *et al.*, 2021). Following the taxonomic key in Knight-Jones & Mackie (2003), we tentatively identified the specimens as *S. japonica.* However, DNA barcoding using the COI gene fragment revealed a genetic distance of 10.6% between specimens from Hong Kong and those of *S. japonica* from Japan, which is comparable to the genetic distance between species of *Sabellastarte* (7%–20% in Capa *et al*. 2010). Therefore, in this paper, this specimen has the provisional species name of *Sabellastarte* sp. h YS-2021. More detailed morphological and molecular analyses should be conducted to describe this potentially distinct species.

This assembly is the first high-quality genome for the genus *Sabellastarte* and one of five genomes available for the family Sabellidae as of March 2026 (data obtained via NCBI datasets, O’Leary *et al.*, 2024).

## Methods

### Sample acquisition

**Figure 1.**
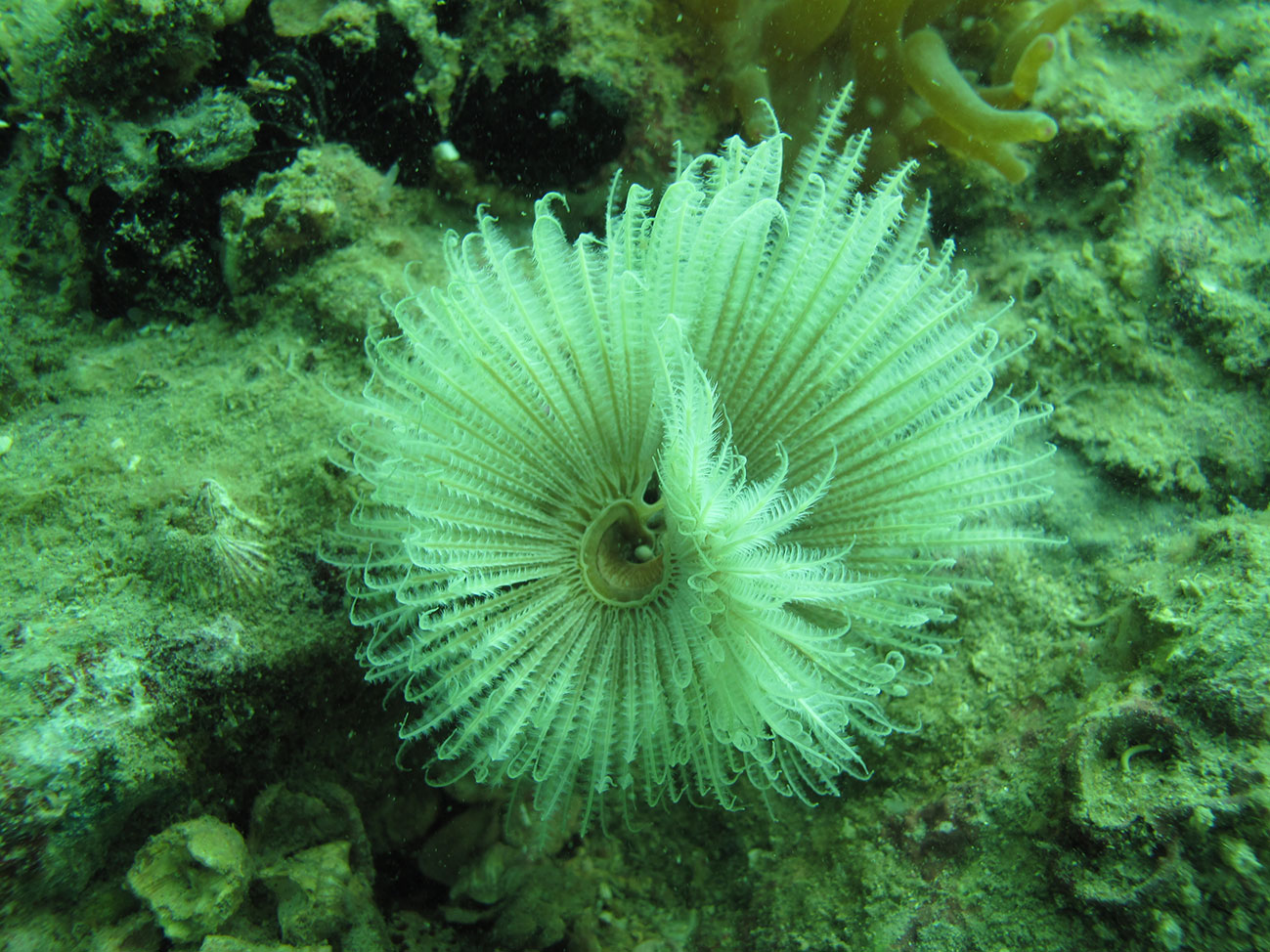
Photograph of the *Sabellastarte* sp. h YS-2021 (wsSabSpea1) specimen used for genome sequencing.

The specimen used for genome sequencing was an adult *Sabellastarte* sp. h YS-2021 (specimen ID QMOUL0000008, ToLID wsSabSpea1; Figure 1), collected from Yau Tong, Hong Kong (latitude 22.2877, longitude 114.2372) on 2021-05-21. The specimen was collected by Keith Kei, identified by Jianwen Qiu, and prepared and dissected by Yanan Sun. The same specimen was used for RNA sequencing.

### Nucleic acid extraction

Protocols for high molecular weight (HMW) DNA extraction developed at the Wellcome Sanger Institute (WSI) Tree of Life Core Laboratory are available on protocols.io ( Howard *et al.*, 2025). The wsSabSpea1 sample was weighed and triaged to determine the appropriate extraction protocol. Tissue from the tentacle was homogenised by cryogenic disruption using the Covaris cryoPREP ^®^ Automated Dry Pulverizer. HMW DNA was extracted using the Manual MagAttract protocol. DNA was sheared into an average fragment size of 12–20 kb following the Megaruptor®3 for LI PacBio protocol. Sheared DNA was purified by manual SPRI (solid-phase reversible immobilisation).

The concentration of the sheared and purified DNA was assessed using a Nanodrop spectrophotometer and Qubit Fluorometer using the Qubit dsDNA High Sensitivity Assay kit. Fragment size distribution was evaluated by running the sample on the FemtoPulse system. For this sample, the final post-shearing DNA had a Qubit concentration of 25.58 ng/μL and a yield of 1 202.26 ng, with a fragment size of 12.8 kb.

RNA was extracted from tentacle tissue of wsSabSpea1 in the Tree of Life Laboratory at the WSI using the RNA Extraction: Automated MagMax™ *mir*Vana protocol. The RNA concentration was assessed using a Nanodrop spectrophotometer and a Qubit Fluorometer using the Qubit RNA Broad-Range Assay kit. Analysis of the integrity of the RNA was done using the Agilent RNA 6000 Pico Kit and Eukaryotic Total RNA assay.

### PacBio HiFi library preparation and sequencing

Library preparation and sequencing were performed at the WSI Scientific Operations core. Libraries were prepared using the SMRTbell Prep Kit 3.0 (Pacific Biosciences, California, USA), following the manufacturer’s instructions. The kit includes reagents for end repair/A-tailing, adapter ligation, post-ligation SMRTbell bead clean-up, and nuclease treatment. Size selection and clean-up were performed using diluted AMPure PB beads (Pacific Biosciences). DNA concentration was quantified using a Qubit Fluorometer v4.0 (ThermoFisher Scientific) and the Qubit 1X dsDNA HS assay kit. Final library fragment size was assessed with the Agilent Femto Pulse Automated Pulsed Field CE Instrument (Agilent Technologies) using the gDNA 55 kb BAC analysis kit.

The sample was sequenced using the Sequel IIe system (Pacific Biosciences, California, USA). The concentration of the library loaded onto the Sequel IIe was in the range 40–135 pM. The SMRT link software, a PacBio web-based end-to-end workflow manager, was used to set-up and monitor the run, and to perform primary and secondary analysis of the data upon completion.

### Hi-C

***Sample preparation and crosslinking***

The Hi-C sample was prepared from 20–50 mg of frozen tentacle tissue of the wsSabSpea1 sample using the Arima-HiC v2 kit (Arima Genomics). Following the manufacturer’s instructions, tissue was fixed and DNA crosslinked using TC buffer to a final formaldehyde concentration of 2%. The tissue was homogenised using the Diagnocine Power Masher-II. Crosslinked DNA was digested with a restriction enzyme master mix, biotinylated, and ligated. Clean-up was performed with SPRISelect beads before library preparation. DNA concentration was measured with the Qubit Fluorometer (Thermo Fisher Scientific) and Qubit HS Assay Kit. The biotinylation percentage was estimated using the Arima-HiC v2 QC beads.

***Hi-C library preparation and sequencing***

Biotinylated DNA constructs were fragmented using a Covaris E220 sonicator and size selected to 400–600 bp using SPRISelect beads. DNA was enriched with Arima-HiC v2 kit Enrichment beads. End repair, A-tailing, and adapter ligation were carried out with the NEBNext Ultra II DNA Library Prep Kit (New England Biolabs), following a modified protocol where library preparation occurs while DNA remains bound to the Enrichment beads. Library amplification was performed using KAPA HiFi HotStart mix and a custom Unique Dual Index (UDI) barcode set (Integrated DNA Technologies). Depending on sample concentration and biotinylation percentage determined at the crosslinking stage, libraries were amplified with 10–16 PCR cycles. Post-PCR clean-up was performed with SPRISelect beads. Libraries were quantified using the AccuClear Ultra High Sensitivity dsDNA Standards Assay Kit (Biotium) and a FLUOstar Omega plate reader (BMG Labtech).

Prior to sequencing, libraries were normalised to 10 ng/μL. Normalised libraries were quantified again to create equimolar and/or weighted 2.8 nM pools. Pool concentrations were checked using the Agilent 4200 TapeStation (Agilent) with High Sensitivity D500 reagents before sequencing. Sequencing was performed using paired-end 150 bp reads on the Illumina NovaSeq 6000.

### RNA library preparation and sequencing

Libraries were prepared using the NEBNext ^®^ Ultra™ II Directional RNA Library Prep Kit for Illumina (New England Biolabs), following the manufacturer’s instructions. Poly(A) mRNA in the total RNA solution was isolated using oligo (dT) beads, converted to cDNA, and uniquely indexed; 14 PCR cycles were performed. Libraries were size-selected to produce fragments between 100–300 bp. Libraries were quantified, normalised, pooled to a final concentration of 2.8 nM, and diluted to 150 pM for loading. Sequencing was carried out on the Illumina NovaSeq 6000, generating paired-end reads.

### Genome assembly

Prior to assembly of the PacBio HiFi reads, a database of *k*-mer counts ( *k* = 31) was generated from the filtered reads using FastK. GenomeScope2 ( Ranallo-Benavidez *et al.*, 2020) was used to analyse the *k*-mer frequency distributions, providing estimates of genome size, heterozygosity, and repeat content.

The HiFi reads were assembled using Hifiasm ( Cheng *et al.*, 2021) with the --primary option. Haplotypic duplications were identified and removed using purge_dups ( Guan *et al.*, 2020). The Hi-C reads ( Rao *et al.*, 2014) were mapped to the primary contigs using bwa-mem2 ( Vasimuddin *et al.*, 2019), and the contigs were scaffolded in YaHS ( Zhou *et al.*, 2023) with the --break option for handling potential misassemblies. The scaffolded assemblies were evaluated using Gfastats ( Formenti *et al.*, 2022), BUSCO ( Manni *et al.*, 2021) and MERQURY.FK ( Rhie *et al.*, 2020).

The mitochondrial genome was assembled using MitoHiFi ( Uliano-Silva *et al.*, 2023).

### Assembly curation

The assembly was decontaminated using the Assembly Screen for Cobionts and Contaminants ( ASCC) pipeline. TreeVal was used to generate the flat files and maps for use in curation. Manual curation was conducted primarily in PretextView and HiGlass ( Kerpedjiev *et al.*, 2018). Scaffolds were visually inspected and corrected as described by Howe *et al*. (2021). Manual corrections included 136 breaks, 102 joins, and removal of 76 haplotypic duplications. This reduced the scaffold count by 37.5%, increased the scaffold N50 by 5.1%, and reduced the total assembly length by 3.5%. The curation process is described at https://gitlab.com/wtsi-grit/rapid-curation . PretextSnapshot was used to generate a Hi-C contact map of the final assembly.

### Assembly quality assessment

The Merqury.FK tool ( Rhie *et al.*, 2020) was run in a Singularity container ( Kurtzer *et al.*, 2017) to evaluate *k*-mer completeness and assembly quality for the primary and alternate haplotypes using the *k*-mer database ( *k* = 31) computed prior to genome assembly. The analysis outputs included assembly QV scores and completeness statistics.

The genome was analysed using the BlobToolKit pipeline, a Nextflow implementation of the earlier Snakemake version ( Challis *et al.*, 2020). The pipeline aligns PacBio reads using minimap2 ( Li, 2018) and SAMtools ( Danecek *et al.*, 2021) to generate coverage tracks. It runs BUSCO ( Manni *et al.*, 2021) using lineages identified from the NCBI Taxonomy ( Schoch *et al.*, 2020). For the three domain-level lineages, BUSCO genes are aligned to the UniProt Reference Proteomes database ( Bateman *et al.*, 2023) using DIAMOND blastp ( Buchfink *et al.*, 2021). The genome is divided into chunks based on the density of BUSCO genes from the closest taxonomic lineage, and each chunk is aligned to the UniProt Reference Proteomes database with DIAMOND blastx. Sequences without hits are chunked using seqtk and aligned to the NT database with blastn ( Altschul *et al.*, 1990). The BlobToolKit suite consolidates all outputs into a blobdir for visualisation. The BlobToolKit pipeline was developed using nf-core tooling ( Ewels *et al.*, 2020) and MultiQC ( Ewels *et al.*, 2016), with containerisation through Docker ( Merkel, 2014) and Singularity ( Kurtzer *et al.*, 2017).

### Metagenome assembly

The metagenome assembly was generated using MetaMDBG ( Benoit *et al.*, 2024) and binned usingMetaBAT2 ( Kang *et al.*, 2019). The resulting bin sets of each binning algorithm were optimised and refined using DAS Tool ( Sieber *et al.*, 2018). PROKKA ( Seemann, 2014) was used to identify tRNAs and rRNAs in each bin, CheckM ( Parks *et al.*, 2015) (checkM_DB release 2015-01-16) was used to assess bin completeness/contamination, and GTDB-Tk ( Chaumeil *et al.*, 2022) (GTDB release 214) was used to taxonomically classify bins. Taxonomic replicate bins were identified using dRep ( Olm *et al.*, 2017) with default settings (95% ANI threshold). All bins were assessed for quality and categorised as metagenome-assembled genomes (MAGs) if they met the following criteria: contamination ≤5%, presence of 5S, 16S, and 23S rRNA genes, at least 18 unique tRNAs, and either ≥90% completeness or ≥ 50% completeness with fully circularised chromosomes ( Bowers *et al.*, 2017). Bins that did not meet these thresholds, or were identified as taxonomic replicates of MAGs, were retained as ‘binned metagenomes’ provided they had ≥50% completeness and ≤10% contamination.

## Genome sequence report

### Sequence data

**Figure 2.**
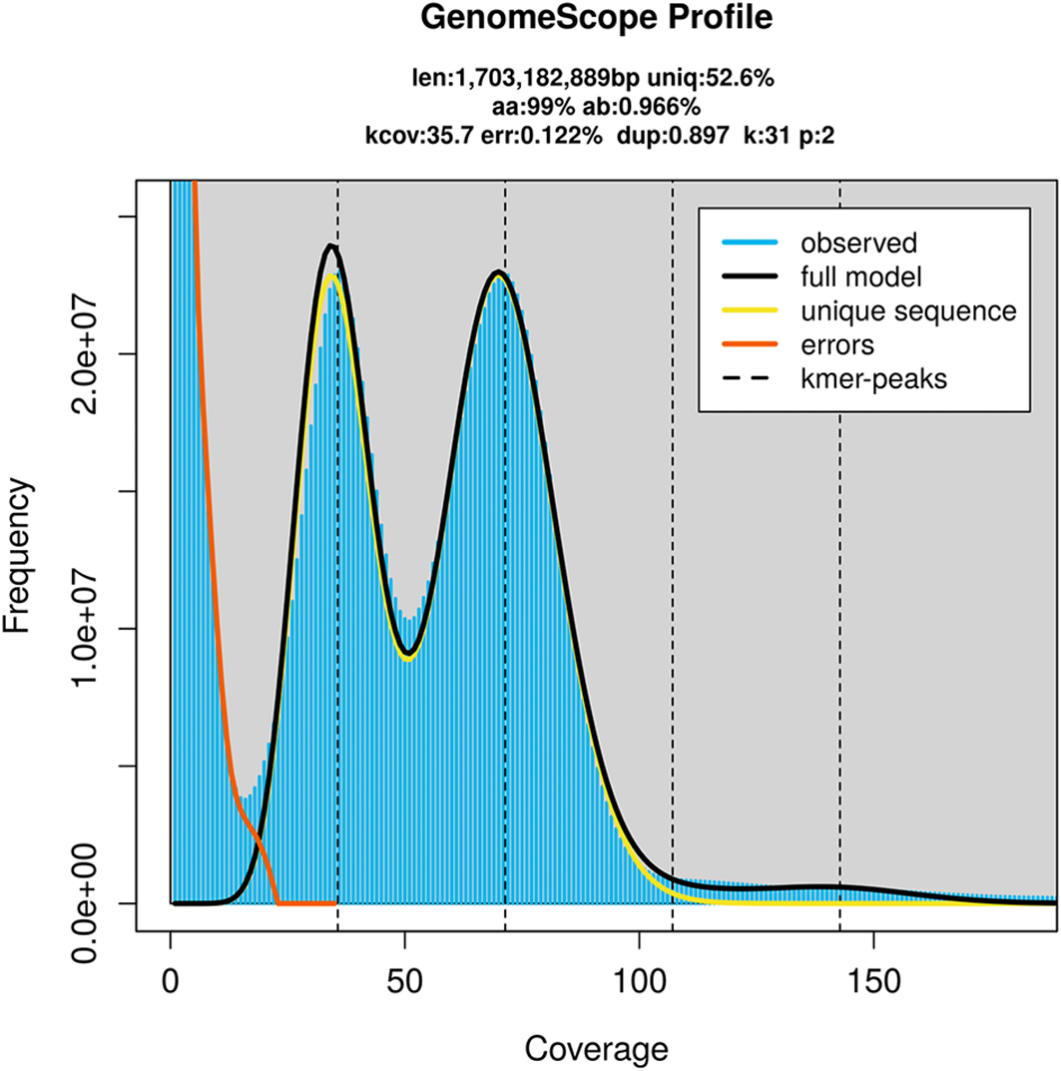
Frequency distribution of *k*-mers generated using GenomeScope2. The plot shows observed and modelled *k*-mer spectra, providing estimates of genome size, heterozygosity, and repeat content based on unassembled sequencing reads.

PacBio sequencing of the *Sabellastarte* sp. h YS-2021 specimen generated 129.26 Gb (gigabases) from 14.21 million reads, which were used to assemble the genome. GenomeScope2.0 analysis estimated the haploid genome size at 1 705.58 Mb, with a heterozygosity of 0.97% and repeat content of 47.42% ( Figure 2). These estimates guided expectations for the assembly. Based on the estimated genome size, the sequencing data provided approximately 48× coverage. Hi-C sequencing produced 206.14 Gb from 682.59 million reads, which were used to scaffold the assembly. RNA sequencing data were also generated and are available in public sequence repositories. Table 1 summarises the specimen and sequencing details.

**Table 1.** Specimen and sequencing data for BioProject PRJEB70268.

Platform	PacBio HiFi	Hi-C	RNA-seq
**ToLID**	wsSabSpea1	wsSabSpea1	wsSabSpea1
**Specimen ID**	QMOUL0000008	QMOUL0000008	QMOUL0000008
**BioSample (source individual)**	SAMEA12097743	SAMEA12097743	SAMEA12097743
**BioSample (tissue)**	SAMEA12097797	SAMEA12097797	SAMEA12097797
**Tissue**	tentacle	tentacle	tentacle
**Instrument**	Sequel IIe	Illumina NovaSeq 6000	Illumina NovaSeq 6000
**Run accessions**	ERR12319377; ERR14749936; ERR12319375; ERR12319376	ERR12318604; ERR12318603	ERR12318605
**Read count total**	14.21 million	682.59 million	37.77 million
**Base count total**	129.26 Gb	206.14 Gb	11.41 Gb

### Assembly statistics

The primary haplotype was assembled, and contigs corresponding to an alternate haplotype were also deposited in INSDC databases. The final assembly has a total length of 1 786.39 Mb in 723 scaffolds, with 364 gaps, and a scaffold N50 of 155.04 Mb ( Table 2).

**Table 2.** Genome assembly data for *Sabellastarte* sp. h YS-2021.

Genome assembly	Primary assembly
**Assembly name**	wsSabSpea1.1
**Assembly accession**	GCA_964017235.1
**Alternate haplotype accession**	GCA_964017245.1
**Assembly level**	chromosome
**Span (Mb)**	1 786.39
**Number of chromosomes**	14
**Number of contigs**	1 087
**Contig N50**	8.99 Mb
**Number of scaffolds**	723
**Scaffold N50**	155.04 Mb
**Organelles**	Mitochondrion: 15.35 kb

**Figure 3.**
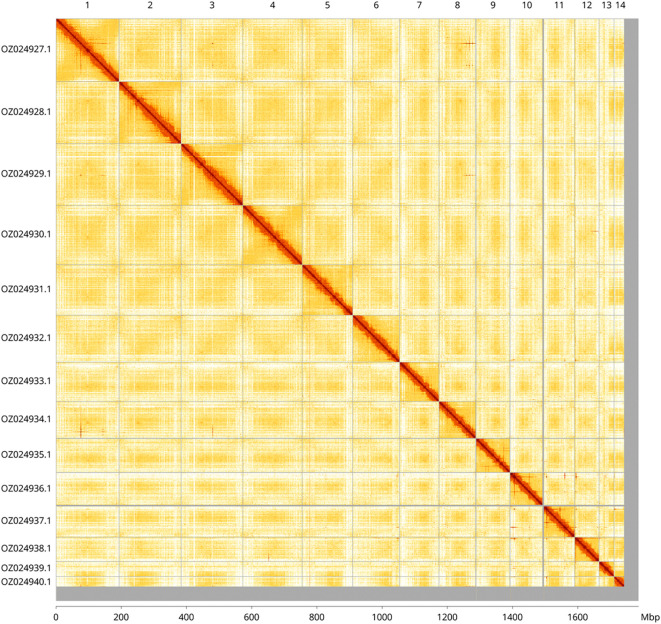
Hi-C contact map of the *Sabellastarte* sp. h YS-2021 genome assembly. Assembled chromosomes are shown in order of size and labelled along the axes, with a megabase scale shown below. The plot was generated using PretextSnapshot.

Most of the assembly sequence (97.94%) was assigned to 14 chromosomal-level scaffolds. These chromosome-level scaffolds, confirmed by Hi-C data, are named according to size ( Figure 3 and Table 3).

**Table 3.** Chromosomal pseudomolecules in the primary genome assembly of *Sabellastarte* sp. h YS-2021 wsSabSpea1.

INSDC accession	Molecule	Length (Mb)	GC%
OZ024927.1	1	193.65	37.50
OZ024928.1	2	190.50	37.50
OZ024929.1	3	189.25	37.50
OZ024930.1	4	181.91	37.50
OZ024931.1	5	155.04	37.50
OZ024932.1	6	143.65	37.50
OZ024933.1	7	120.81	37
OZ024934.1	8	113.46	37.50
OZ024935.1	9	104.61	37
OZ024936.1	10	102.92	37.50
OZ024937.1	11	94.87	37
OZ024938.1	12	74.81	37
OZ024939.1	13	46.04	36.50
OZ024940.1	14	38	38

The mitochondrial genome was also assembled (length 15.35 kb, OZ024941.1). This sequence is included as a contig in the multifasta file of the genome submission and as a standalone record.

### Assembly quality metrics

**Figure 4.**
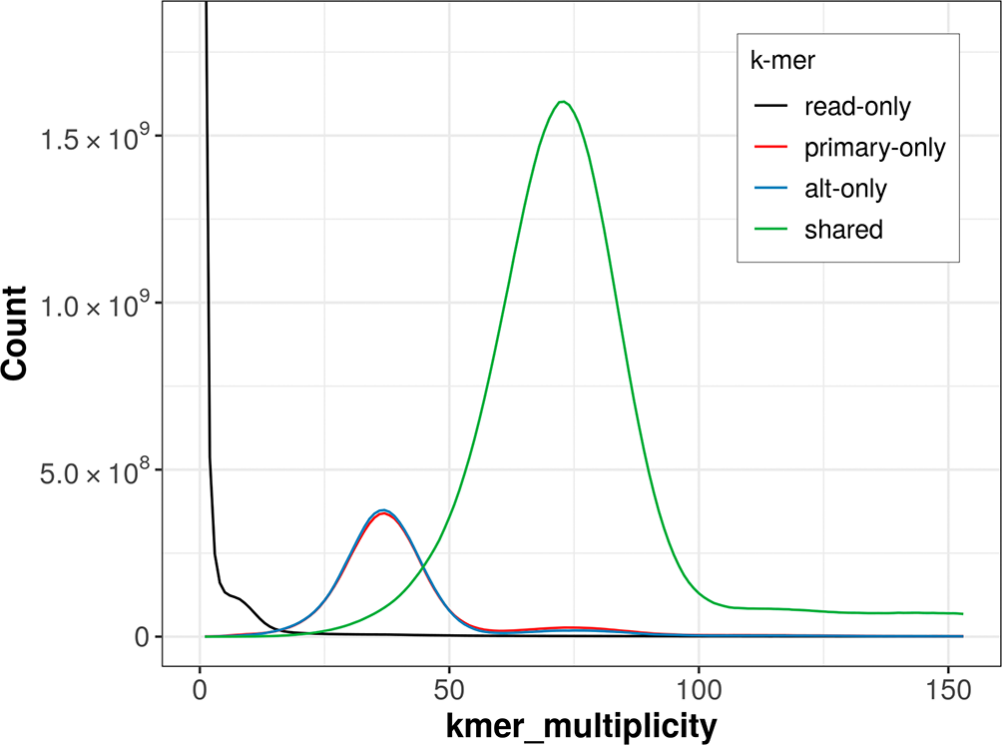
Evaluation of *k*-mer completeness using MerquryFK. This plot illustrates the recovery of *k*-mers from the original read data in the final assemblies. The horizontal axis represents *k*-mer multiplicity, and the vertical axis shows the number of *k*-mers. The black curve represents *k*-mers that appear in the reads but are not assembled. The green curve corresponds to *k*-mers shared by both haplotypes, and the red and blue curves show *k*-mers found only in one of the haplotypes.

The combined primary and alternate assemblies achieve an estimated QV of 63.1. The *k*-mer completeness is 81.95% for the primary assembly, 81.97% for the alternate haplotype, and 99.07% for the combined assemblies ( Figure 4).

**Figure 5.**
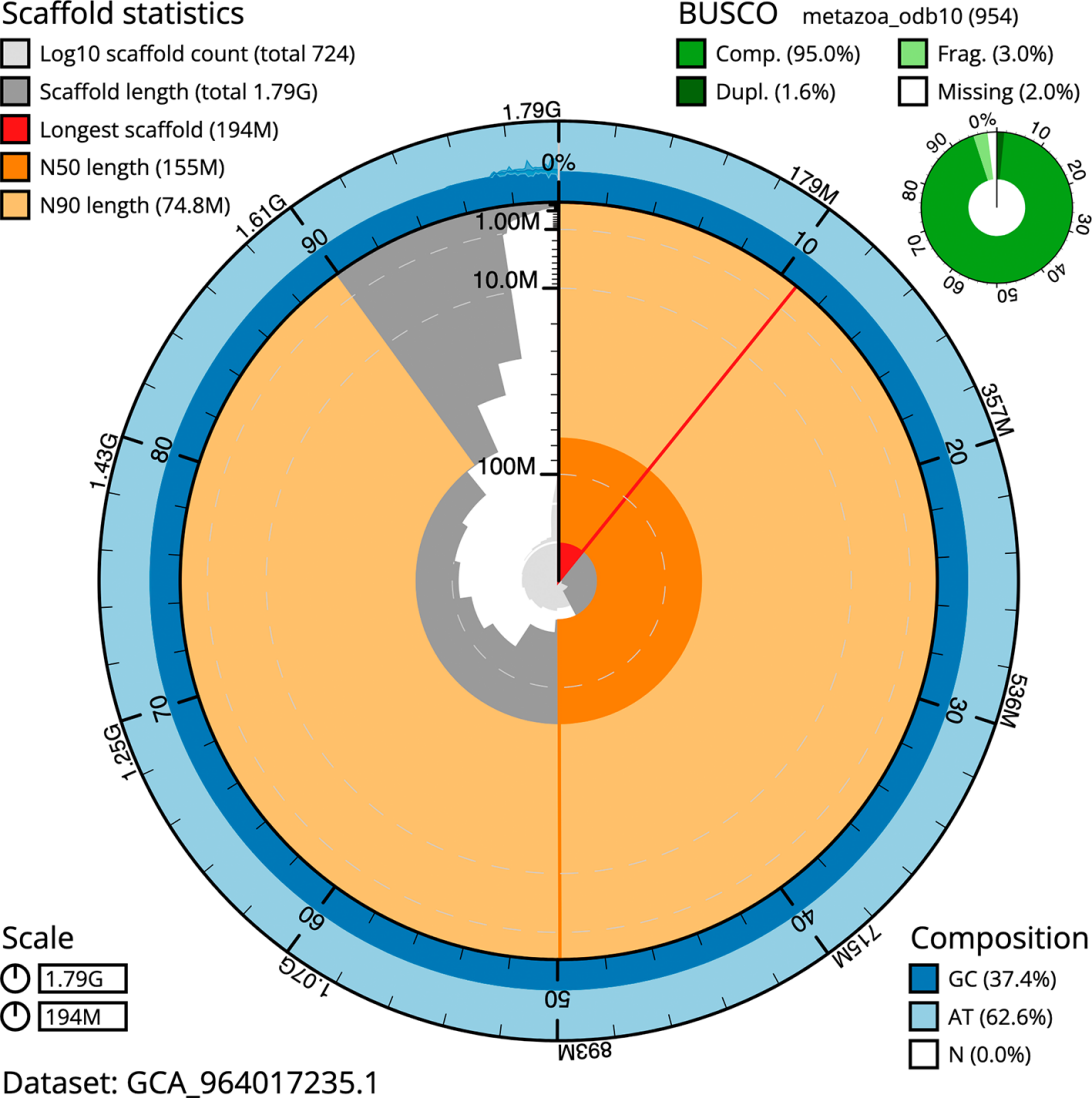
Assembly metrics for wsSabSpea1.1. The BlobToolKit snail plot provides an overview of assembly metrics and BUSCO gene completeness. The circumference represents the length of the whole genome sequence, and the main plot is divided into 1 000 bins around the circumference. The outermost blue tracks display the distribution of GC, AT, and N percentages across the bins. Scaffolds are arranged clockwise from longest to shortest and are depicted in dark grey. The longest scaffold is indicated by the red arc, and the deeper orange and pale orange arcs represent the N50 and N90 lengths. A light grey spiral at the centre shows the cumulative scaffold count on a logarithmic scale. A summary of complete, fragmented, duplicated, and missing BUSCO genes in the metazoa_odb10 set is presented at the top right. An interactive version of this figure can be accessed on the BlobToolKit viewer.

**Figure 6.**
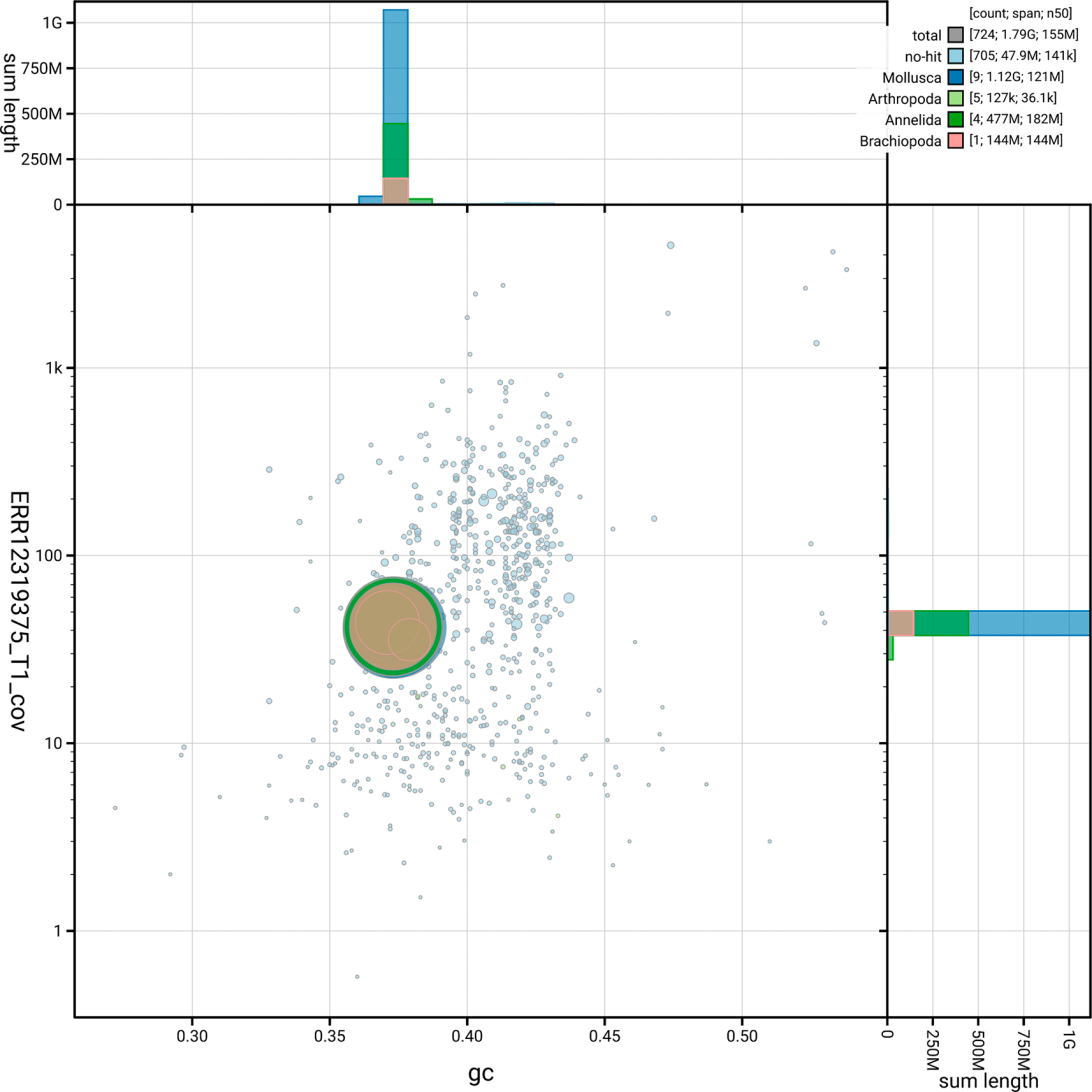
BlobToolKit blob plot for wsSabSpea1.1. The plot shows base coverage (vertical axis) and GC content (horizontal axis). The circles represent scaffolds, with the size proportional to scaffold length and the colour representing phylum membership. The histograms along the axes display the total length of sequences distributed across different levels of coverage and GC content. An interactive version of this figure is available on the BlobToolKit viewer.

BUSCO v.5.5.0 analysis using the metazoa_odb10 reference set ( *n* = 954) identified 95.0% of the expected gene set (single = 93.4%, duplicated = 1.6%). The snail plot in Figure 5 summarises the scaffold length distribution and other assembly statistics for the primary assembly. The blob plot in Figure 6 shows the distribution of scaffolds by GC proportion and coverage.

Table 4 lists the assembly metric benchmarks adapted from Rhie *et al.* (2021) and the Earth BioGenome Project Report on Assembly Standards. The EBP metric, calculated for the primary assembly, is **6.C.Q62**, meeting the recommended reference standard.

**Table 4.** Earth Biogenome Project summary metrics for the *Sabellastarte* sp. h YS-2021 assembly.

Measure	Value	Benchmark
EBP summary (primary)	6.C.Q62	6.C.Q40
Contig N50 length	8.99 Mb	≥ 1 Mb
Scaffold N50 length	155.04 Mb	= chromosome N50
Consensus quality (QV)	Primary: 62.3; alternate: 64.1; combined: 63.1	≥ 40
*k*-mer completeness	Primary: 81.95%; alternate: 81.97%; combined: 99.07%	≥ 95%
BUSCO	C:95.0% [S:93.4%; D:1.6%]; F:3.0%; M:2.0%; n:954	S > 90%; D < 5%
Percentage of assembly assigned to chromosomes	97.94%	≥ 90%

*Note:* EBP summary uses log10(Contig N50); chromosome-level (C) or log10(Scaffold N50); Q (Merqury QV). BUSCO: C = complete; S = single-copy; D = duplicated; F = fragmented; M = missing; n = orthologues BUSCO v5.5.0 lineage: metazoa_odb10.

### Metagenome report

We recovered five bins from the metagenome assembly, of which one met the criteria for MAGs, a fully circularised genome. The recovered bins represented two bacterial phyla, with genome sizes ranging from 0.90 to 1.84 Mbp (mean: 1.37 ± 0.36 Mbp). Mean completeness was 54.8% (± 22.4%) with 1.6% (± 1.7%) contamination. Table 5 summarises the taxa and quality of the metagenome bins.

**Table 5.** Quality metrics and taxonomic assignments of the binned metagenomes.

Taxon	Taxid	GTDB taxonomy	Quality	Size (bp)	Contigs	Circular	Mean cov.	Compl.(%)	Contam.(%)
Spirochaetota bacterium	2202144	UBA12135	Low	896 009	5	No	5.45	32.40	2.80
*Endozoicomonas* sp.	1892382	Gammaproteobacteria	Low	1 738 037	96	No	2.45	29.89	4.44
Gammaproteobacteria bacterium	1913989	Gammaproteobacteria	Medium	1 247 572	3	No	9.24	52.49	0.38
*Aquella* sp.	2593688	Gammaproteobacteria	Medium	1 139 531	8	No	5.12	70.99	0.61
Spirochaetia bacterium	2053615	Spirochaetia	High	1 835 469	1	Yes	31.52	88.37	0

**Table 6.** Software versions and sources.

Software	Version	Source
**BEDTools**	2.30.0	https://github.com/arq5x/bedtools2
**BLAST**	2.14.0	ftp://ftp.ncbi.nlm.nih.gov/blast/executables/blast+/
**BlobToolKit**	4.3.9	https://github.com/blobtoolkit/blobtoolkit
**BUSCO**	5.5.0	https://gitlab.com/ezlab/busco
**bwa-mem2**	2.2.1	https://github.com/bwa-mem2/bwa-mem2
**Cooler**	0.8.11	https://github.com/open2c/cooler
**DIAMOND**	2.1.8	https://github.com/bbuchfink/diamond
**fasta_windows**	0.2.4	https://github.com/tolkit/fasta_windows
**FastK**	1.1	https://github.com/thegenemyers/FASTK
**Gfastats**	1.3.6	https://github.com/vgl-hub/gfastats
**GenomeScope2.0**	2.0.1	https://github.com/tbenavi1/genomescope2.0
**Hifiasm**	0.19.5-r587	https://github.com/chhylp123/hifiasm
**HiGlass**	1.13.4	https://github.com/higlass/higlass
**MerquryFK**	1.1.2	https://github.com/thegenemyers/MERQURY.FK
**Minimap2**	2.24-r1122	https://github.com/lh3/minimap2
**MitoHiFi**	3	https://github.com/marcelauliano/MitoHiFi
**MultiQC**	1.14; 1.17 and 1.18	https://github.com/MultiQC/MultiQC
**Nextflow**	23.04.1	https://github.com/nextflow-io/nextflow
**PretextSnapshot**	0.0.5	https://github.com/sanger-tol/PretextSnapshot
**PretextView**	1.0.3	https://github.com/sanger-tol/PretextView
**Seqtk**	1.3	https://github.com/lh3/seqtk
**Singularity**	3.9.0	https://github.com/sylabs/singularity
**sanger-tol/ascc**	0.1.0	https://github.com/sanger-tol/ascc
**sanger-tol/blobtoolkit**	0.4.0	https://github.com/sanger-tol/blobtoolkit
**sanger-tol/curationpretext**	1.4.2	https://github.com/sanger-tol/curationpretext
**TreeVal**	1.4.0	https://github.com/sanger-tol/treeval
**YaHS**	1.2a.2	https://github.com/c-zhou/yahs

## Author information

Contributors are listed at the following links:

• Members of the Wellcome Sanger Institute Tree of Life Management, Samples and Laboratory team
• Members of Wellcome Sanger Institute Scientific Operations – Sequencing Operations
• Members of the Wellcome Sanger Institute Tree of Life Core Informatics team
• Members of the EBI Aquatic Symbiosis Genomics Data Portal Team
• The Aquatic Symbiosis Genomics Project leadership

## Wellcome Sanger Institute – Legal and Governance

The materials that have contributed to this genome note have been supplied by a Tree of Life collaborator. The Wellcome Sanger Institute employs a process whereby due diligence is carried out proportionate to the nature of the materials themselves, and the circumstances under which they have been/are to be collected and provided for use. The purpose of this is to address and mitigate any potential legal and/or ethical implications of receipt and use of the materials as part of the research project, and to ensure that in doing so we align with best practice wherever possible.

The overarching areas of consideration are:

• Ethical review of provenance and sourcing of the material
• Legality of collection, transfer and use (national and international)

Each transfer of samples is undertaken according to a Research Collaboration Agreement or Material Transfer Agreement entered into by the Tree of Life collaborator, Genome Research Limited (operating as the Wellcome Sanger Institute) and in some circumstances other Tree of Life collaborators.

## Data Availability

European Nucleotide Archive: Sabellastarte sp. h YS-2021 (feather duster worm). Accession number PRJEB70268. The genome sequence is released openly for reuse. The *Sabellastarte* sp. h YS-2021 genome sequencing initiative is part of the Aquatic Symbiosis Genomics Project (PRJEB43743) and the Sanger Institute Tree of Life Programme (PRJEB43745). All raw sequence data and the assembly have been deposited in INSDC databases. The genome will be annotated using available RNA-Seq data and presented through the Ensembl pipeline at the European Bioinformatics Institute. Raw data and assembly accession identifiers are reported in Tables 1 and 2. Production code used in genome assembly at the WSI Tree of Life is available at https://github.com/sanger-tol . Table 6 lists software versions used in this study.
