# Supplementary material for: The chromosomal genome sequence of the feather duster worm, Sabellastarte sp. h YS-2021 (Sabellida: Sabellidae) and its associated microbial metagenome sequences
Source: Wellcome Open Res. 2026 May 11;11:274. [Version 1] doi: 10.12688/wellcomeopenres.26503.1 (PMC13234546; doi:10.12688/wellcomeopenres.26503.1)
Supplement: Peer review history

# Reviewer report: Reviewer response for version 1

**DOI:** 10.21956/wellcomeopenres.29188.r155505

**Published:** 2026-06-03

**Authors:** Thomas D. Lewin

**Affiliations:** 1 School of Biological and Behavioural Sciences, Queen Mary University of London (Ringgold ID: 4617), London, England, UK

Sun et al. present a reference assembly for the feather duster worm *Sabellastarte*. The genome note is of the standard format produced by the Tree of Life Programme. Methods and protocols are very well documented and the genome is extremely high quality. I have only a couple of queries for the authors to address.

 First, regarding the BlobToolKit output in Figure 6: if I am reading it correctly, only four of the 724 scaffolds have a phylum membership of Annelida while 9 are Mollusca, 5 Arthropoda and 1 Brachiopoda. I expect this kind of result with very understudied phyla that may be unrepresented in databases but I am quite surprised to see it for an annelid (though I have now seen it for annelids on several occasions).  Do the authors have an explanation for this result? Are there not enough annelids in the reference database? If this is the source of the issue, should the database not be updated? Either way, this result must be explained in the text, because taking this plot at face value one could question whether the assembly really is of an annelid at all (though I do not doubt that it is!).

 Second, regarding the same Figure, it is clear that many of the small scaffolds with unusual coverage and GC content and no hits are contaminants. Were these removed from the assembly as part of the metagenome assembly process? This is currently not clear. If so, would it be possible to include another blob plot for the final assembly?

 Finally, a problem I have repeatedly raised with this style of note is the complete absence of information regarding genome annotation, which I think should be included for transparency.

 Minor points

 Abstract – acronym MAG needs full explanation at first use.

 Abstract and throughout text – rogue space in numbers where a comma should be e.g. 1 786.39 megabases

Are sufficient details of methods and materials provided to allow replication by others?

Yes

Is the rationale for creating the dataset(s) clearly described?

Yes

Are the datasets clearly presented in a useable and accessible format?

Yes

Are the protocols appropriate and is the work technically sound?

Yes

Reviewer Expertise:

Evolutionary genomics

I confirm that I have read this submission and believe that I have an appropriate level of expertise to confirm that it is of an acceptable scientific standard.

# Reviewer report: Reviewer response for version 1

**DOI:** 10.21956/wellcomeopenres.29188.r155504

**Published:** 2026-05-28

**Authors:** Jun Kim

**Affiliations:** 1 Chungnam National University, Daejeon, Daejeon, South Korea

This manuscript represents an important contribution by providing information on the genome assembly of an individual belonging to the genus Sabellastarte, potentially representing a novel species, together with its associated metagenome assembly. I believe that this study is valuable in that it demonstrates the potential to examine organismal characteristics at the genome level, and to conduct species-level analyses based on genomic information, even when detailed morphological and conventional molecular characterization may be limited.

 In the future, comparative analyses with genome assemblies of closely related taxa, such as Sabellastarte japonica, may further help establish genomic approaches for species identification across a broader range of organisms. Such approaches may, in some respects, parallel the way in which prokaryotic species are often identified or delineated based on genome sequence divergence. Overall, the manuscript is well written, and I have only a few minor comments that I hope will help improve its clarity and completeness.

 1. Clarification of tentacle tissue used for different experiments

 The manuscript states:

 “Tissue from the tentacle was homogenised by cryogenic disruption using the Covaris cryoPREP® Automated Dry Pulverizer. HMW DNA was extracted”

 “RNA was extracted from tentacle tissue of wsSabSpea1”

 “The Hi-C sample was prepared from 20–50 mg of frozen tentacle tissue of the wsSabSpea1”

 It would be helpful to clarify whether the “tentacle tissue” mentioned in these different contexts refers to the same tentacle sample or to different tentacles from the same individual. Providing this information would improve the reproducibility and interpretability of the sample preparation procedures.

 2. Details on the data used for metagenome assembly

 The manuscript states:

 “The metagenome assembly was generated using MetaMDBG (Benoit et al., 2024) and binned using MetaBAT2 (Kang et al., 2019).”

 I recommend providing additional details on how the input data for the metagenome assembly were obtained. For example, it would be useful to clarify whether the metagenome assembly was generated from HiFi reads that were co-sequenced as contaminants during host genomic DNA sequencing, possibly originating from microorganisms present on the surface of the tentacles, or whether a separate sample, such as intestinal or fecal material from the wsSabSpea1 individual, was used to generate an independent HiFi dataset for metagenomic assembly.

 At present, information regarding DNA extraction and library preparation specifically for the metagenome assembly appears to be lacking. Adding these details would make the metagenomic component of the study clearer and more reproducible.

 3. Assessment of telomeric repeats and potential mis-scaffolding

 The manuscript states:

 “Most of the assembly sequence (97.94%) was assigned to 14 chromosomal-level scaffolds. These chromosome-level scaffolds, confirmed by Hi-C data, are named according to size (Figure 3 and Table 3).”

 It would be useful to report how many telomeric repeat clusters were detected at chromosomal ends. In addition, I suggest assessing whether any telomeric repeat clusters are located internally within chromosome-level scaffolds, as such patterns could indicate potential mis-scaffolding events.

 Including this information would further support the accuracy of the chromosome-level assembly and would help readers evaluate whether the Hi-C scaffolding process introduced any structural errors.

 4. Interpretation of k-mer-based QV and completeness estimates

 The manuscript states:

 “The combined primary and alternate assemblies achieve an estimated QV of 63.1. The k-mer completeness is 81.95% for the primary assembly, 81.97% for the alternate haplotype, and 99.07% for the combined assemblies (Figure 4).”

 Because the same raw reads appear to have been used both for genome assembly and for the k-mer-based quality assessment, it would be helpful to mention that the estimated QV may be relatively high for this reason. In other words, when the sequencing reads used for assembly are also used for k-mer-based validation, the resulting QV estimate may partly reflect consistency with the input data rather than fully independent validation of assembly accuracy.

 Adding a brief note on this point would provide a more cautious and transparent interpretation of the reported QV and completeness values.

Are sufficient details of methods and materials provided to allow replication by others?

Yes

Is the rationale for creating the dataset(s) clearly described?

Yes

Are the datasets clearly presented in a useable and accessible format?

Yes

Are the protocols appropriate and is the work technically sound?

Yes

Reviewer Expertise:

Genomics

I confirm that I have read this submission and believe that I have an appropriate level of expertise to confirm that it is of an acceptable scientific standard.
